# Glycosylation signature of plasma IgA of critically ill COVID-19 patients

**DOI:** 10.3389/fimmu.2024.1439248

**Published:** 2024-10-24

**Authors:** Daniel P. Potaczek, Bianca D. M. van Tol, David Falck, Christina Krolczik, Kristina Zlatina, Wilhelm Bertrams, Jochen Wilhelm, Bernd Schmeck, Benjamin Seeliger, Sascha David, Chrysanthi Skevaki, Elisabeth Mack, Werner Seeger, Liliana Schaefer, Sebastian P. Galuska, Manfred Wuhrer, Małgorzata Wygrecka

**Affiliations:** ^1^ Center for Infection and Genomics of the Lung, Universities of Giessen and Marburg Lung Center, German Center for Lung Research, Giessen, Germany; ^2^ Translational Inflammation Research Division & Core Facility for Single Cell Multiomics, Medical Faculty, Philipps-University Marburg, Marburg, Germany; ^3^ Bioscientia MVZ Labor Mittelhessen GmbH, Giessen, Germany; ^4^ Center for Proteomics and Metabolomics, Leiden University Medical Center, Leiden, Netherlands; ^5^ Research Institute for Farm Animal Biology (FBN), Dummerstorf, Germany; ^6^ Institute for Lung Research, Universities of Giessen and Marburg Lung Center, German Center for Lung Research, Philipps-University Marburg, Marburg, Germany; ^7^ Institute for Lung Health, Justus-Liebig University, German Center for Lung Research, Giessen, Germany; ^8^ Department of Respiratory Medicine, Hanover Medical School, Hanover, Germany; ^9^ Biomedical Research in End-Stage and Obstructive Lung Disease, Hannover Medical School, German Center for Lung Research, Hannover, Germany; ^10^ Institute of Intensive Care, University Hospital Zurich, Zurich, Switzerland; ^11^ Institute of Laboratory Medicine, Philipps-University Marburg, Marburg, Germany; ^12^ Department of Hematology, Oncology and Immunology, Philipps-University Marburg, Marburg, Germany; ^13^ Department of Internal Medicine II, Universities of Giessen and Marburg Lung Center, German Center for Lung Research, Giessen, Germany; ^14^ Institute of Pharmacology and Toxicology, Goethe University, Frankfurt am Main, Germany

**Keywords:** ARDS, COVID-19, glycosylation, immunoglobulin A, NETosis

## Abstract

Thromboembolic complications are common in severe COVID-19 and are thought to result from excessive neutrophil-extracellular-trap (NET)-driven immunothrombosis. Glycosylation plays a vital role in the efficiency of immunoglobulin A (IgA) effector functions, with significant implications for NET formation in infectious diseases. This study represents the first comprehensive analysis of plasma IgA glycosylation during severe SARS-CoV-2 or Influenza A infection, revealing lower sialylation and higher galactosylation of IgA1 O-glycans in acute respiratory distress syndrome (ARDS), regardless of the underlying cause of the disease. Importantly, N-glycans displayed an infection-specific pattern, with N47 of IgA2 showing diminished sialylation and bisection, and N340/N327 of IgA1/2 demonstrating lower fucosylation and antennarity along with higher non-complex glycans in COVID-19 compared to Influenza. Notably, COVID-19 IgA possessed strong ability to induce NET formation and its glycosylation patterns correlated with extracellular DNA levels in plasma of critically ill COVID-19 patients. Our data underscores the necessity of further research on the role of IgA glycosylation in the modulation of pathogen-specific immune responses in COVID-19 and other infectious diseases.

## 1 Introduction

Thromboembolic complications are common in severe COVID-19 and are thought to result from aberrant immunothrombosis. This host defense mechanism is used to prevent pathogen dissemination. It links inflammation and coagulation and involves different cell types including endothelial cells, monocytes, platelets and neutrophils. Uncontrolled immunothrombosis can result in vessel occlusion and tissue damage (1, 2). Neutrophils play a crucial role in this process by releasing neutrophil extracellular traps (NETs) during a regulated form of cell death, known as NETosis. NETs are composed of large, chromosomal DNA fibers decorated with histones and a variety of microbicidal peptides and proteolytic enzymes. The main physiological role of NETs is to trap and kill invading pathogenes, however, NETs may also contribute to exacerbated activation of platelets and coagulation factors, thereby promoting thrombosis (1, 2). Although NETosis has been reported in various infectious diseases (3), it has received specific attention in the context of COVID-19-associated coagulopathy. Accordingly, NET-rich occlusions of small pulmonary vessels have been reported in lungs of patients who died from COVID-19 (1, 2). Additionally, SARS-CoV-2-specific immunoglobulin A (IgA) levels were found to correlate with the amounts of extracellular DNA (exDNA, a marker of NET formation) in plasma of critically ill COVID-19 patients (4).

Several studies have shown that glycosylation plays a vital role in the efficacy of IgA effector functions, with significant implications in autoimmune and infectious diseases (5–11). Specifically, the glycosylation signature of IgA has been linked to complement activation and systemic inflammation status (12–14). However, while glycomic studies of IgA have demonstrated a correlation between glycosylation profiles of IgA and the pathomechanisms of diseases such as IgA nephropathy or rheumatoid arthritis (7–11), the glycosylation signatures of plasma IgA obtained from patients with severe COVID-19 remained largely unexplored. Given the capacity of IgA to trigger NET formation and the significance of NETosis in COVID-19-associated coagulopathy (15), the present study hypothesized that the COVID-19 IgA glycosylation patterns differ from the glycosylation profiles of IgA isolated from the plasma of patients with acute respiratory distress syndrome (ARDS) caused by Influenza A virus and that this partially explains high rates of NETosis in COVID-19.

## 2 Materials and methods

### 2.1 Study population

The study included 28 individuals, comprising 10 critically ill patients diagnosed with COVID-19 [COVID-19; WHO score 5-7 (16)], 8 patients with ARDS caused by Influenza A virus (Influenza), and 10 healthy volunteers (Healthy). Patient samples were taken within 2 days after onset of ARDS. Investigations were approved by the ethical review boards of Philipps-University of Marburg (no.: 57/20), Hannover Medical School (no.: 8146_BO_K_2018), and Justus-Liebig-University of Giessen (no.: 05/00). Written informed consent was obtained from all participants or their next-of-kin. The demographic information of the participants is presented in **Table 1**.

**Table 1:** Demographic and clinical characteristics of the patients.

	Healthy controls (n=10)	COVID-19 ARDS (n=10)	Influenza ARDS (n=8)
Age, years	68 [41-76]	68 [39-82]	66 [42-85]
Male sex, n	8	8	7
Body mass index, kg/m^2^	–	30 [25-40]	32 [22-53]
28-day mortality, n	–	3 (30)	3 (38)
^1^ICU-LOS, days	–	34 [19-48]	28 [18-48]
Ventilation, days	–	26 [13-39]	17 [2-66]
^2^ECMO, n	–	1	5
Anticoagulant (heparin), n		10	8
Comorbidities Diabetes	–	2	1
Hypertension	–	3	3

^1^ICU-LOS, intensive care unit length of stay; ^2^ECMO, extracorporeal membrane oxygenation. Median [minimum-maximum] is shown.

### 2.2 Isolation of plasma IgA and NET formation

Plasma IgA was purified by affinity chromatography as previously described (7). Silver staining of polyacrylamide gels and a pyrochrome assay (VWR International, Darmstadt, Germany) were used to assess IgA purity and endotoxin contamination, respectively. Neutrophils were isolated from male and female healthy donors (n=5) using the density gradient separation method (7). For NET formation assay, neutrophils (10^5^ cells/well in 250 µL RPMI 1640 medium without phenol red) were seeded on bovine serum albumin-precoated coverslips in a 24-well culture plate (Sarstedt, Nümbrecht, Germany). After addition of 10 μg/mL IgA or 200 nM phorbol 12-myristate 13-acetate (PMA, a positive control), the cells were incubated for 90 min at 37°C and 5% CO_2_. Subsequently, the neutrophils were fixed for 30 min at 37°C by adding 250 µL of 8% paraformaldehyde (Merck Millipore, Burlington, MA) directly to the wells and then washed twice with phosphate-buffered saline (PBS). The coverslips were mounted with Vectashield Mounting Medium containing 4’,6-diamidyno-2-fenyloindol (DAPI; Vector Laboratories, Burlingame). Images were taken with a Nikon Eclipse Ci-L fluorescent microscope (Nikon Instruments, Tokyo, Japan). NET formation was determined as the percentage of cells undergoing NETosis relative to the total number of cells. The levels of exDNA were measured in supernatants or plasma using Quant-iT-PicoGreen dsDNA Assay Kit (Thermo Fisher Scientific, Waltham, MA).

### 2.3 Analysis of IgA glycosylation

For glycosylation analysis, total plasma IgA was isolated with CaptureSelect IgA Affinity Matrix (Thermo Fisher Scientific), denatured, reduced and alkylated. IgA (glyco)peptides were generated by trypsin digestion and subjected to high-throughput nano-liquid chromatography-mass spectrometry (nanoLC-MS)-based method enabling determination of IgA glycosylation profiles in a protein- and site-specific manner, as described previously (6).

## 3 Results

**Figure 1:**
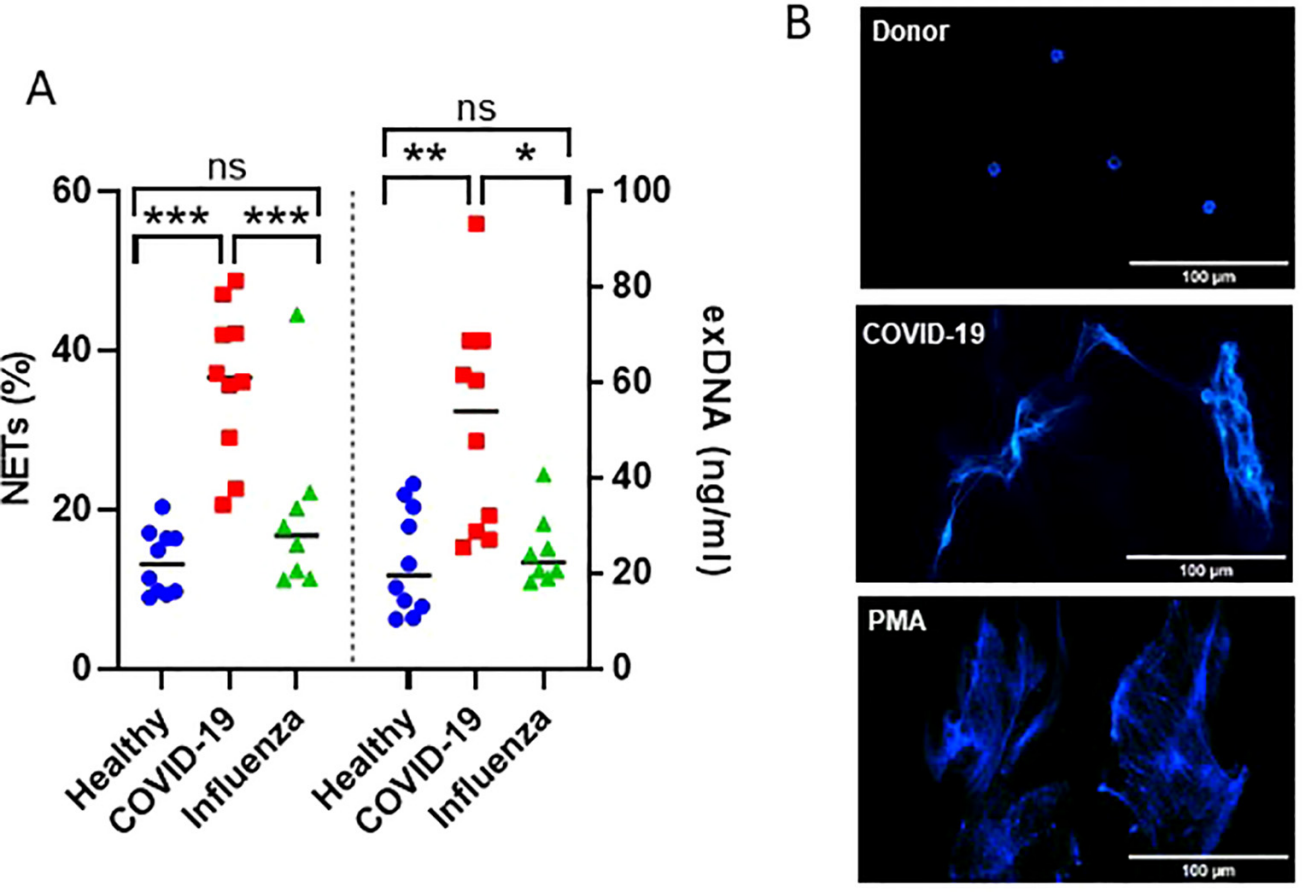
IgA from plasma of COVID-19 patients triggers NET formation. **(A)** NET formation following incubation of neutrophils with IgA isolated from plasma of healthy subjects, severe COVID-19, or ARDS Influenza patients. The percentage of cells undergoing NETosis relative to the total number of cells and exDNA measured in cell culture media are shown. Values were analyzed in R 4.3.0 by linear models. Comparisons of group means were performed using the function glht from the package multcomp 1.4. p-values were adjusted to allow the control of the family-wise error rate across all pairwise comparisons using the single-step method. ***p<0.001; **p<0.01; *p<0.05; ns, not significant. **(B)** Representative fluorescence microscopy images showing NET formation upon stimulation of neutrophils with IgA isolated from plasma of healthy subjects or severe COVID-19 patients. PMA was used as a positive control. DNA stained with DAPI is shown.

**Figure 2:**
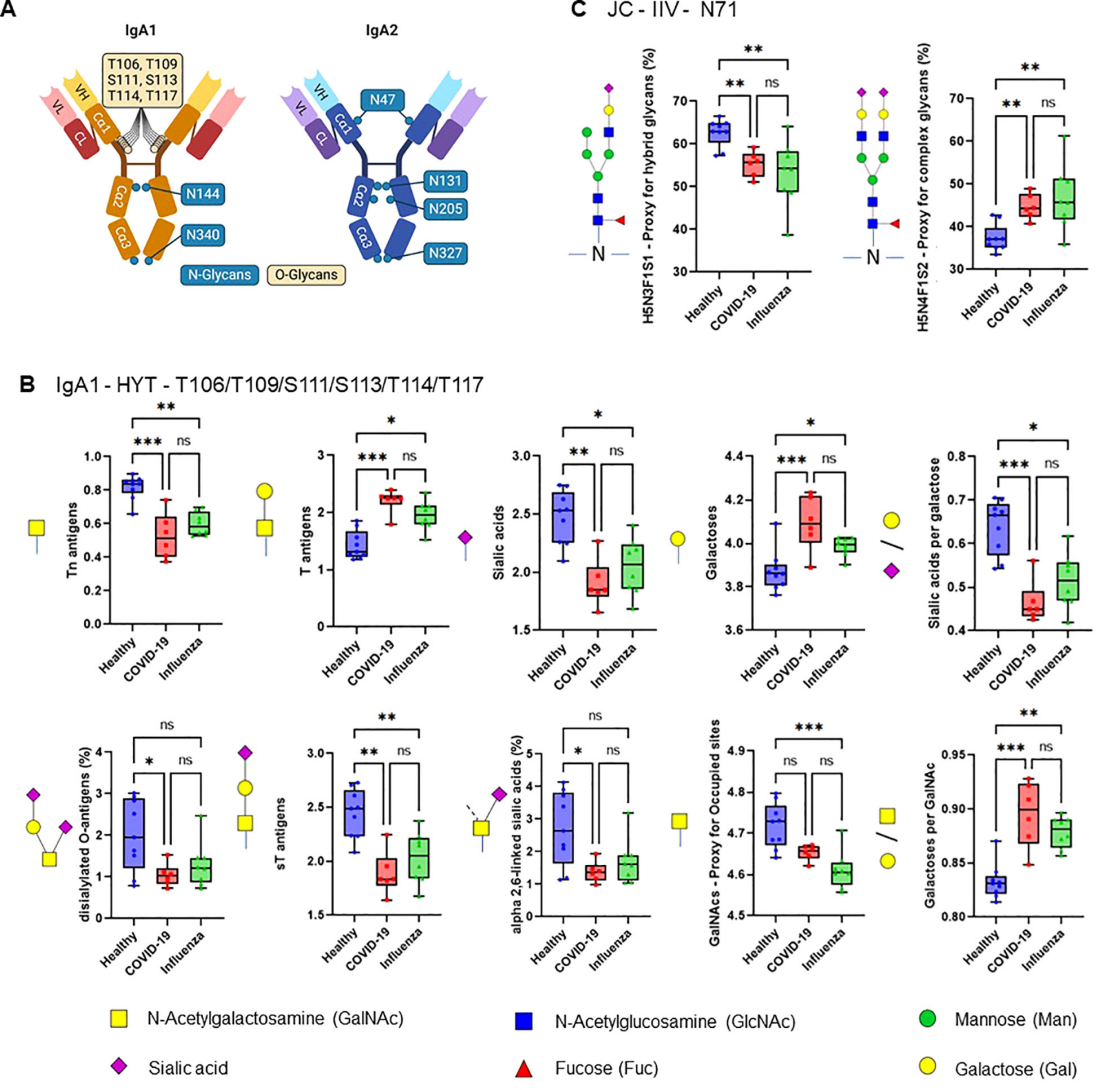
ARDS IgA glycosylation patterns differ from those of healthy controls. **(A)** Schematic representation of plasma IgA1/2. IgA has two heavy (H) chains and two light (L) chains connected by disulphide bridges. The heavy chains are composed of three constant (Cα) domains and one variable (V) domain, and the light chains are composed of one C domain and one V domain. Locations of known IgA N- and O-glycans are shown. Created with biorender.com. **(B, C)** Differences in plasma IgA glycosylation profiles between healthy and patients with COVID-19 or Influenza ARDS for indicated clusters and glycosylation sites (**B**, IgA1 - HYT - T106/T109/S111/S113/T114/T117; **C**, JC - IIV - N71). Differences in plasma IgA glycosylation patterns were assessed using the Kruskal-Wallis test followed by uncorrected Dunn’s test. ***p<0.001; **p<0.01; *p<0.05; ns, not significant.

**Figure 3:**
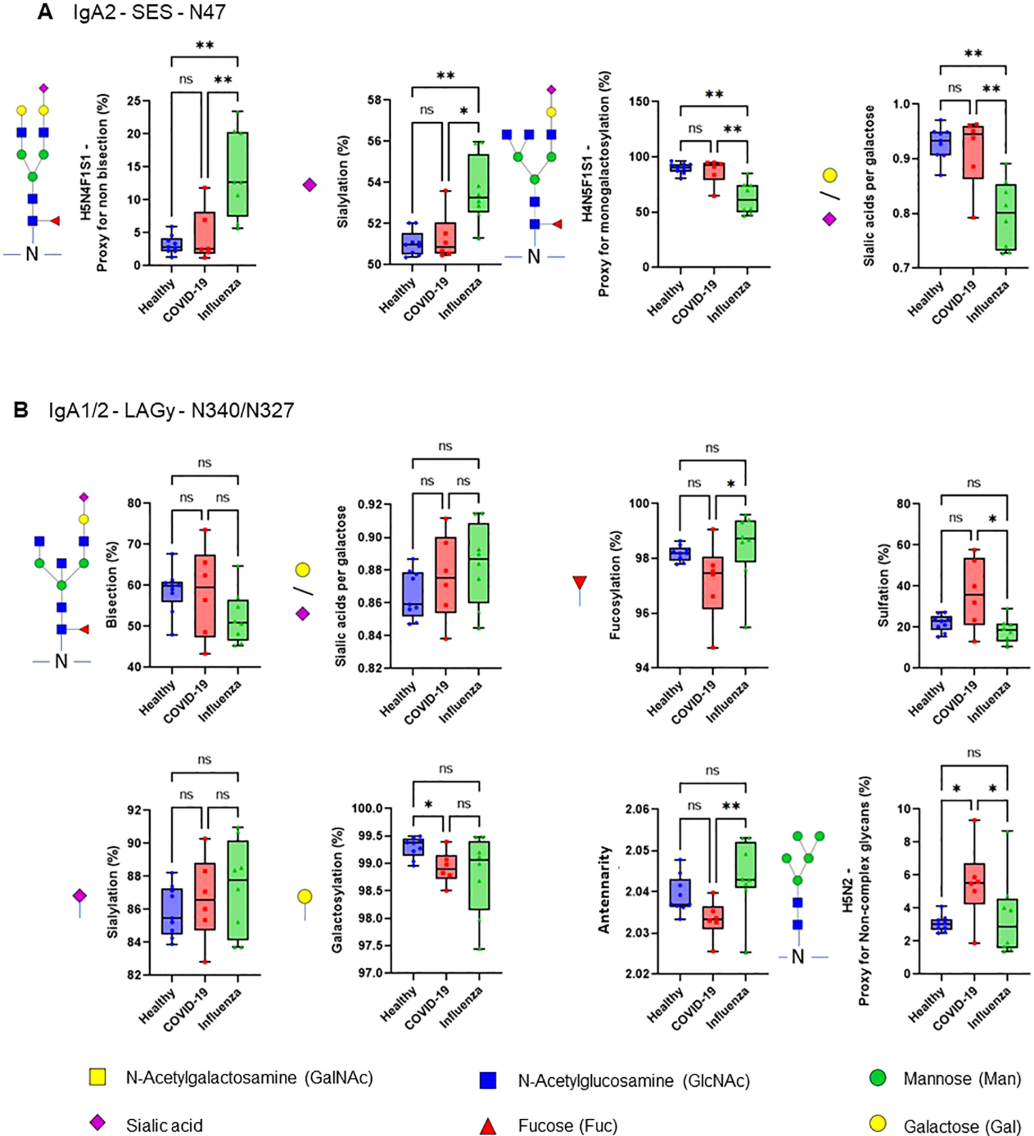
COVID-19 IgA glycosylation patterns differ from those of ARDS Influenza. **(A, B)** Differences in plasma IgA glycosylation profiles between healthy and patients with COVID-19 or Influenza ARDS for indicated clusters and glycosylation sites (**A**, IgA2 - SES - N47; **B**, IgA1/2 - LAGy - N340/N327). **p<0.01; *p<0.05; ns, not significant. Differences in plasma IgA glycosylation patterns were assessed using the Kruskal-Wallis test followed by uncorrected Dunn’s test.

The efficiency of the purified IgA was evaluated based on its ability to induce NET formation. COVID-19 IgA demonstrated a strong ability to induce NETosis, as evidenced by a significant increase in NET formation and exDNA release (**Figure 1A**). Representative images of NETs formed upon incubation of neutrophils with COVID-19 IgA or PMA, used as a positive control, are shown in **Figure 1B**. Subsequently, the glycome of IgA isolated from patients with COVID-19 and Influenza, as well as from healthy subjects, was analyzed. An overview of IgA glycosylation sites is given in **Figure 2A**. Both IgA1 and IgA2 carry multiple conserved N-glycosylation sites. In addition, IgA1 has up to six O-glycosylation sites in the hinge region (HYT) that can carry antigens, Tn (N-acetylgalactosamine (GalNAc)-), T (galactose (Gal)-GalNAc-), or their sialylated forms (sialyl-Tn (sTn) and sialyl-T (sT)) (5–7, 12). IgA monomers can be covalently linked to a J-chain (JC), forming dimers and higher oligomers. However, most serum IgA is monomeric and IgA1 is the dominant subclass (5, 7). The nanoLC-MS data revealed significant alterations in the O-glycosylation pattern of IgA1, with elevated levels of T antigen, Gal content, and galactosylation per GalNAc in ARDS as compared to healthy subjects. In contrast, Tn and sT antigens, sialylation per Gal, disialylated O-antigens, the levels of α2,6-linked sialic acid residues, and the overall sialic acid content were lower in ARDS as opposed to healthy individuals (**Figure 2B**). Regarding N-glycosylation, COVID-19 and Influenza ARDS patients showed diminished levels of a monosialylated hybrid-type glycan and elevated levels of a disialylated complex N-glycans at N71 in the JC (**Figure 2C**). In addition, N-glycans at N47 of IgA2 showed higher levels of non-bisection and sialylation and less monogalactosylation and sialic acid per Gal in Influenza than in COVID 19 patients (**Figure 3A**). The N340/N327 glycosylation sites, shared between IgA subclasses, showed more fucosylation and antennarity, but lower levels of sulfation and non-complex glycans in Influenza as opposed to COVID-19 group (**Figure 3B**). To obtain insights into relationship between COVID-19 exDNA plasma levels and IgA glycosylation patterns, the correlation analysis was performed. Positive correlations (correlation coefficient (r)>0.8) were found between exDNA levels and sialylation in IgA2-SES and IgA1-HYT and negative correlations (r<-0.8) were detected between exDNA levels and monogalactosylation in IgA2-SES and T antigen in IgA1-HYT.

## 4 Discussion

Infectious disease-associated shifts in IgG glycosylation profiles have been broadly described, suggesting the controlled modulation of the immune responses by O- and/or N-glycans. For example, anti-spike protein IgG1 afucosylation was associated with enhanced activation of immune cells (17) and disturbed hemostasis (18). This study represents first comprehensive analysis of plasma IgA glycosylation during active severe infections caused by SARS-CoV-2 or Influenza A, revealing lower sialylation and higher galactosylation of IgA1 O-glycans in patients with ARDS, regardless of the underlying cause of the disease. Importantly, N-glycans displayed an infection-specific signature, with N47 of IgA2 showing elevated sialylation and bisection and N340/N327 of IgA1/2 demonstrating higher fucosylation and antennarity along with lower non-complex glycans in Influenza as compared to COVID-19. The consequences of these unique IgA glycosylation patterns for the induction of host defense processes are not known and require further research. However, in view of existing data, it is tempting to speculate that distinct glycosylation profiles of COVID-19 IgA may contribute to the aberrant immunothrombosis and thus to thromboembolic complications, frequently observed in severe COVID-19 (19). It should be noted that apparent glycosylation differences in the shared IgA1/2 sites could hint toward corresponding alterations in the subclass ratio, which may also impact IgA effector functions (7). Either notion is supported by our data, showing enhanced NETosis in the presence of COVID-19 IgA and correlations between COVID-19 exDNA plasma levels and IgA glycosylation patterns.

This study has several limitations. While this work presents a significant observation, it is based on a relatively low number of participants (predominantly males) with wide age range, thus the findings necessitate further validation in well-balanced cohorts. Furthermore, the data presented herein are based on single time-point measurements, precluding the examination of dynamic changes. In addition, despite endeavoring to match patient cohorts as closely as possible, the influence of ECMO, duration of mechanical ventilation, and comorbidities on IgA glycosylation in Influenza versus COVID-19 groups cannot be ruled out. Lastly, the limited amounts of plasma restricted the evaluation of antigen-specific IgA, thereby constraining the ability to determine the relationship between the IgA glycosylation signature and disease causality, along with thrombotic complications.

Our data underscores the necessity and significance of further research on the role of IgA glycosylation in the modulation of pathogen-specific immune responses with the aim of designing effective targeted intervention strategies that focus on antibody glycosylation in COVID-19 and other infection diseases.

## Data Availability

The LC-MS IgA glycosylation data have been deposited to the ProteomeXchange Consortium via the PRIDE (20) partner repository with the dataset identifier PXD057019.
